# Retraction: Specific Transfection of Inflamed Brain by Macrophages: A New Therapeutic Strategy for Neurodegenerative Diseases

**DOI:** 10.1371/journal.pone.0314537

**Published:** 2024-12-17

**Authors:** The PLOS ONE Editors

Following publication of this article [1, 2] an investigation by the University of North Carolina at Chapel Hill concluded that research misconduct occurred. Specifically, the investigation found falsification of research results as follows:

- In the corrected version of Figure 3B [2], the research results in the first panel (*i*.*c*. PBS followed by *i*.*v*. injection of PBS) originated from an unrelated experiment.
- In Figure 3B [1, 2], the research results in the third panel from the left (*i*.*c*. 6-OHDA followed by *i*.*v*. injection of PBS) originated from an unrelated experiment.

In light of the concerns about the reliability and integrity of the reported results the *PLOS ONE* Editors retract this article.

The corresponding author EVB responded but did not confirm agreement nor disagreement with the editorial decision, and indicated that the wrong data were included in the article in error. EVB stands by the article’s findings and apologizes for the issues with the published article. MJH, YZ, EBH, VM, SA, ZH, PS, SDH, NLK, RLM, HEG, and AVK agreed with the retraction. MJH, EBH, RLM, HEG, and AVK apologize for the issues with the published article. MJH, RLM, and HEG stand by the article’s findings.

At the time of retraction of [1], the Correction [2] was republished to amend the author list.
